# Individualized alignment in total knee arthroplasty using image-based robotic assistance: Video article

**DOI:** 10.1007/s00132-018-3637-1

**Published:** 2018-09-14

**Authors:** Tilman Calliess, Max Ettinger, Peter Savov, Roman Karkosch, Henning Windhagen

**Affiliations:** Department for Orthopedic Surgery at Annastift Hospital, Hannover Medical School, Anna-von-Borries-Str. 1–7, 30625 Hannover, Germany

**Keywords:** Robotic assisted, Image based, Kinematic alignment, Total knee arthroplasty, No releases, Roboter-assistiert, Bildgestützt, Kinematisches Alignment, Knietotalprothese, No Releases

## Abstract

**Introduction:**

Over the past decades many innovations were introduced in total knee arthroplasty (TKA) focusing on implant longevity and higher procedural precision; however, there are still a high number of dissatisfied patients. It was reported that better anatomical alignment may result in improved patient outcome; however, current technologies have limitations to achieve this. The aim of this video article is to describe the technique of individualized alignment in TKA with the use of image-based robotic assistance.

**Methods:**

The technology is based on an individual patient knee model computed from segmented computed tomography (CT) scans. A preoperative planning of prosthesis position is conducted following the principle of kinematic alignment. Intraoperatively the soft tissue envelope is recorded and the computer predicts the gap balance based on the virtual planning. The prosthesis position is then adapted to achieve balanced gaps and to avoid soft tissue release. This technique is shown in a cadaver operation and clinical examples of two patients are described.

**Results:**

With the combination of anatomically oriented prosthesis positioning and minor adaptations with respect to the soft tissue, an individualized alignment is achieved with reduced need of soft tissue release. The robotic-assisted surgery guarantees a precise implementation of the planning. The initial experience showed a promising outcome in short-term follow-up.

**Video online:**

The online version of this article (10.1007/s00132-018-3637-1) contains a video on patient individualized alignment in total knee arthroplasty. The article and video are available in the electronic full text archive at SpringerMedizin.de under http://www.springermedizin.de/der-orthopaede. The video can be found at the end of the article as supplementary material.

## Zusammenfassung

**Einleitung:**

In den letzten Jahrzehnten wurden viele Innovationen in der Knieendoprothetik eingeführt, mit dem Ziel einer höheren Operationspräzision und längeren Implantatstandzeit; dennoch gibt es immer noch viele unzufriedene Patienten nach Knieersatz. In der aktuellen Literatur wird berichtet, dass eine anatomischere Ausrichtung zu einem besseren klinischen Outcome führen kann, doch es fehlt aktuell an Technologien mit denen diese Konzepte reproduzierbar umsetzbar sind. Ziel dieses Videobeitrags ist es, die Technik der individualisierten Ausrichtung einer Knietotalendoprothese mithilfe bildbasierter Roboterunterstützung zu beschreiben.

**Methoden:**

Das Verfahren basiert auf einem individuellen Kniemodell, das aus einem Computertomogramm (CT) segmentiert wird. Die präoperative Planung der Prothesenposition erfolgt nach dem Prinzip der kinematischen Ausrichtung. Intraoperativ wird die Bandspannung erfasst. Darauf basierend wird die resultierende Weichteilbalancierung vorhergesagt. Nunmehr kann der Chirurg durch Feinanpassung der Prothesenposition die perfekte Balancierung des Kniegelenkes herstellen und somit weitgehend auf Releases verzichten. Dieses Konzept wird anhand einer Kadaveroperation gezeigt, und 2 klinische Beispiele werden beschrieben.

**Ergebnisse:**

Durch die Kombination von anatomisch orientierter Prothesenpositionierung und Anpassungen entsprechend der individuellen Weichgewebssituation wird eine individuelle Prothesenausrichtung erreicht. Die robotergestützte Operationstechnik ermöglicht die präzise Umsetzung der Planung. Erste Erfahrungen zeigen im kurzfristigen Verlauf bereits vielversprechende Ergebnisse.

## Introduction

There is currently a widespread consensus that the optimal alignment in total knee arthroplasty (TKA) is with reference to the mechanical axis of the limb, correcting any deformities to neutral and defining component position with respect to a theoretical mechanical ideal; however, it has been shown that a neutral alignment is physiological only for a minority of patients with the average having a constitutional varus alignment by 1.2° [3]. There is also a broad variation in the individual anatomy regarding the physiological overall limb alignment and also in the joint line obliquity, femoral flexion, tibial slope and shape of the articular surfaces.

Following the idea of mechanical alignment with the established standard surgical techniques and prosthetic designs, there are still a high number of dissatisfied patients after TKA [14]. Recently, different approaches to reduce the number of dissatisfied patients have been discussed and investigated. These included the introduction of computer assistance during surgery to improve the procedural precision and reproducibility. The second discussion was about a more physiological and individual alignment of the components to reduce paradoxical knee motion and soft tissue imbalances.

Computer-assisted surgery was introduced into hospitals in the late 1990s in the form of passive navigation systems [18]. Today, there is good evidence that computer navigation results in higher precision in component positioning compared to manual instrumentation in the context of total knee arthroplasty (TKA), especially reducing alignment outliers to the mechanical axis [9, 12, 15]. Recently, reduced revision rates were reported as a result of the more accurate surgical procedure [1]; however, it could not be shown that this precision resulted in superior outcome or less dissatisfied patients [4, 8]. It is discussed as one potential reason that the current concepts of navigated TKA still lack consideration of the individual situation of the patient. With mainly imageless systems available, the distinct evaluation of the patient physiological anatomy is challenging. On the other hand, there is growing evidence that a more anatomical and individual alignment may improve patient outcome after TKA, especially in varus patients [2, 21]. The idea of an individual patient alignment is most consistently implemented in the concept of kinematic alignment (KA) [6, 16] where the implant components are positioned individually to restore the pre-arthritic surface anatomy of the knee focusing on the distal and posterior femoral condyles. The tibial alignment is adjusted to the femur with respect to the soft tissue balance. Thus, the patient’s constitutional alignment is re-established three dimensionally. In the literature several studies have shown a clinical benefit of KA over mechanical alignment (MA) in TKA [7, 10, 11, 17]; however, some treatment failures have also been reported. Most of these outliers were attributed to the inaccuracy of current surgical techniques to achieve KA [5, 6] and it is known that increasing the physiological tibial varus or slope might lead to an overload of the bone interface or early implant loosening [19, 26, 28].

There are therefore some reasonable arguments to follow the idea of a patient individual alignment in TKA; however, the solely anatomical approach in KA lacks the inclusion of the individual soft tissue situation, plus a high accuracy is required to conduct this virtual hybrid plan precisely during surgery. A technology combining these characteristics is the image-based semi-automated robotic-assisted surgery systems that were very recently introduced for TKA (2016). The focus of this video article is to describe the general concept of the hybrid technique to achieve patient individualized alignment in TKA on a cadaveric example. Additionally, two clinical cases are described and initial clinical evidence is discussed.

## Surgical technique

Both cadaveric and clinical cases described here were operated on using the semi-automated haptic Stryker MAKO® robotic system (Stryker, Kalamazoo, MI, USA) with the TKA application together with the Stryker Triathlon® knee.

### Preoperative planning

Prior to surgery a computed tomography (CT) scan of the hip, knee and ankle is performed according to the manufacturer’s standard protocol. This is uploaded to the proprietary software platform and segmented to an individual three-dimensional model of the knee. Based on this, an initial preoperative planning is conducted following the principle of KA for restoring the original natural femoral surface. This means that the distal and posterior femoral resection levels are set to 7 mm bone resection independently from the resulting alignment angle. Therefore, the physiological valgus of the distal femur is restored as well as the rotational alignment with reference to the posterior condylar line (PCL). As it is a CT-based planning, cartilage thickness was calculated to 1 mm, taking the elasticity of the cartilage and meniscus into account. If necessary, femoral size is adapted to best fit the sagittal profile (anterior offset and condyle radii) and flexion of the distal femur. If flexion of the component is changed to avoid notching or anterior lift-off, the pivot point is set to the transepicondylar axis (TEA) so as not to affect the mid-flexion stability. The only limitation in sizing is not to produce a mediolateral overhang.

**Fig. 1:**
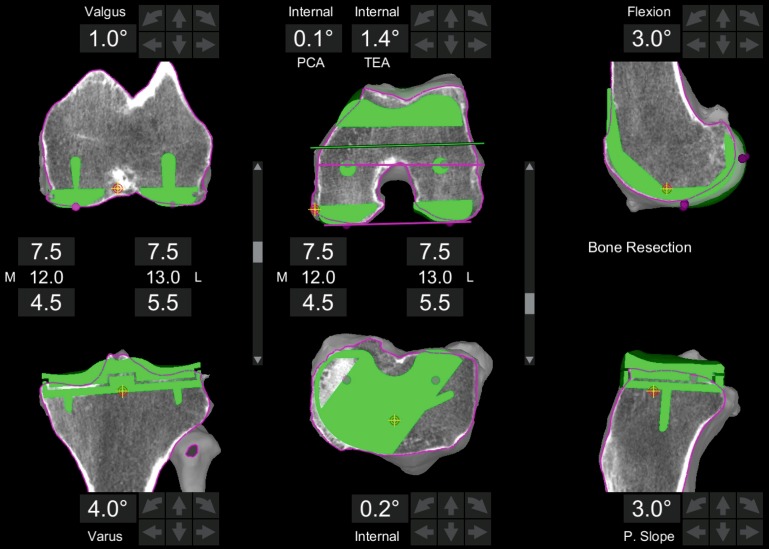
Screenshot of a prosthetic preplanning based on a patient individual knee model from the CT scan. *Left* is the frontal, in the *middle* the axial and on the *right* the sagital CT view with the prosthesis displayed in *green*. Prosthetic alignment is based on the principle of kinematic alignment with symmetric 7.5 mm resection on the distal and dorsal femur. The tibia is aligned to the surface of the proximal jointline. The resulting component orientation is outputted in degrees to the mechanical axes

The tibial varus-valgus alignment is pre-set to a tangent to the medial and lateral tibial plateau. A varus angulation of a maximum of 4° is allowed. The rotational alignment is kept to the anteroposterior axis of the plateau. The tibial slope is orientated to the natural slope and reduced by 2–3° to compensate for the resection of the ACL. If the slope is changed the pivot point is set to the posterior third of the plateau, where the ideal biomechanical contact point of the prosthesis is meant to be, to minimize the effect on the flexion stability of the knee. The tibial resection level is pre-set to a maximum of 7 mm laterally or 5 mm medially. A clinical example of a preplanning is displayed in Fig. 1.

### Set-up and bone referencing

The robotic system is set up and calibrated according to the standard protocol. Tibial and femoral trackers are fixed to the bones and a standard parapatellar incision to the knee is carried out that follows the bone registration according to the standard protocol capturing 40 random points on the surface of each bone. As it is an image-based system, bone registration is made with a sharp probe to guarantee bone contact but capturing the cartilage surface or soft tissue coverage. The required overall accuracy of the bone registration is defined below 0.5 mm deviation. Osteophytes are then removed and the ACL is sacrificed.

### Recording of soft tissue envelope

Next is the recording of the soft tissue situation. With the use of lollipop spacer blocks according to unilateral knee arthroplasty, the collateral ligaments are broad to tension trying to replicate the physiological length of the ligaments with no overstrain. In the case of unilateral osteoarthritis or in mild wear situations a manual varus-valgus stress is applied. The resulting gaps are captured at full extension and 90° flexion and measured in millimeters.

### Balancing algorithm with adapting prosthesis position

The prosthesis position is adjusted to achieve symmetrically balanced gaps and to avoid or minimize soft tissue release as described in more detail below. The target for the optimal gap balance is a perfectly symmetrical extension gap at 18–19 mm, depending on the individual laxity (8 mm distal femur component +9 mm tibia component with smallest onlay). In the flexion gap the target is to have the same balance and gap width in the medial compartment, whereas a physiological lateral laxity up to 21 mm is acceptable. Also, it is acceptable if the flexion gap is 1 mm tighter than the extension because usually it is technically more difficult to correctly record the flexion gap, tending to capture it tighter than it actually is.

**Fig. 2:**
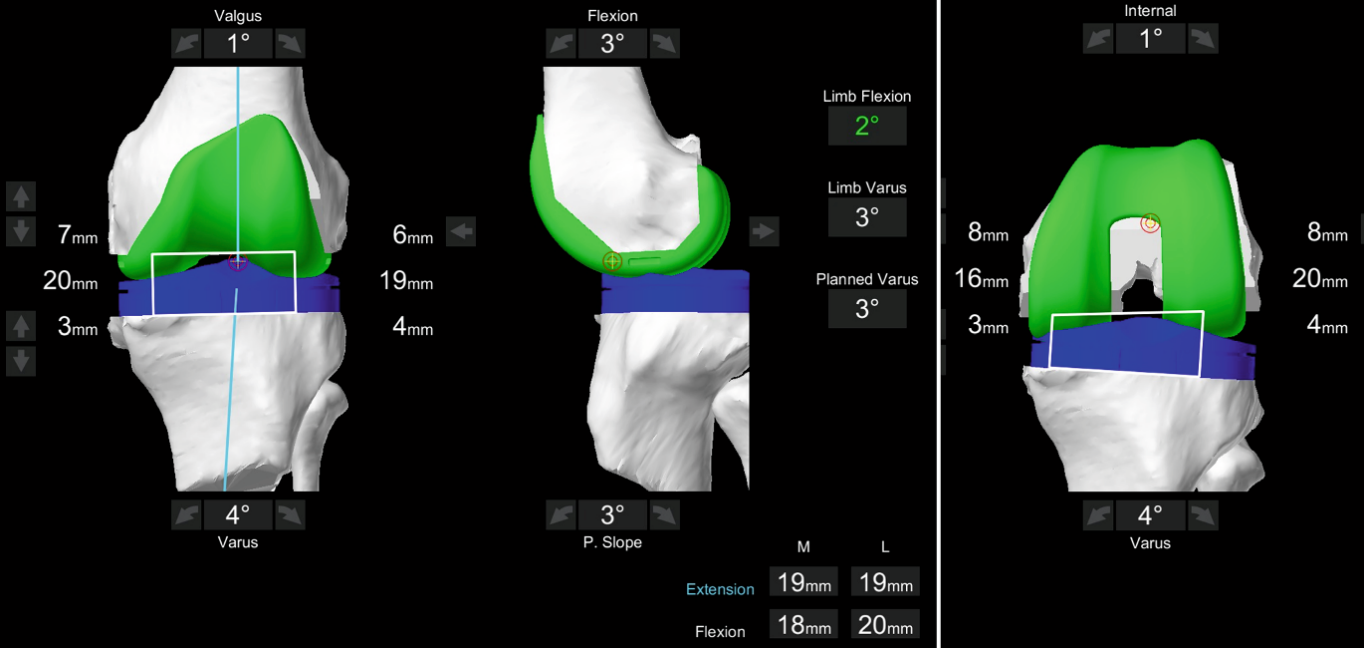
Screenshot of adapted prosthetic position based on soft tissue information. The resection levels and gaps are displayed in mm, as well as the overall limb alignment. The predicted resulting gaps are displayed in the *lower right corner*

The first parameter to address for balancing is always the tibial cut because the basic philosophy of individualized alignment is to closely restore the femoral anatomy. Thus, distal and posterior femoral resections are critical for adaptations because alterations in these parameters massively affect knee kinematics. If a gap asymmetry requires adaptation (unlikely in the concept of kinematic alignment), the femoral cuts are limited to a maximum of 9 mm (maximum 1 mm difference to prosthesis thickness). An alternative target for imbalanced gaps is the tibial slope; however, the physiological slope should not be exceed. If there would be still mismatch between the flexion and extension gaps, for example, the distal femoral resection is reduced first, instead of increasing the posterior cut thickness. If the resulting gaps are then to small, the tibial resection level is lowered. In varus cases the varus-valgus orientation or rotation of the femur is adapted for balancing the gaps with currently a resulting overall varus alignment up to 3–4° is acceptable. If there is still an imbalance present and the resulting alignment would be beyond 4° off the mechanical axis, a controlled soft tissue release is carried out until a symmetrical extension gap is achieved. This is again recorded into the system and after that the flexion gap is adapted via changing the femoral rotation. In Fig. 2 the soft tissue adapted planning is displayed as an example.

### Cutting the bone

**Fig. 3:**
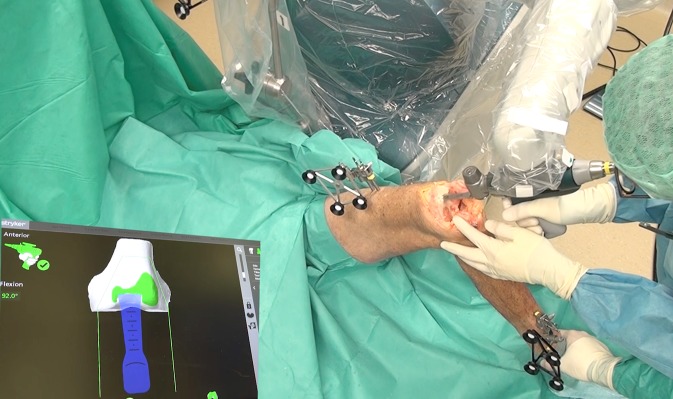
Screenshot from video displaying the cutting process with the robotic device. The robotic arm defines the cutting plane and sets the boundaries for the saw; however, the saw is operated by the surgeon

When the prosthetic position is optimized to achieve the desired gap balance and alignment, the resulting hybrid virtual plan is conducted by the use of the haptic robotic-assisted system. The robotic system defines the cutting plane and only allows the surgeon to move the saw within the given boundaries (see Fig. 3). When the surgeon is about to leave that zone the system will counteract or simply stop the saw blade (see video). During surgery accurate bone cuts as well as the final position of the trial prosthesis are verified using the integrated computer navigation. With the Stryker Monogram Balancer®, as well as with the trial components the planned gap size and balance is re-evaluated and recorded as well as the resulting alignment and range of motion. If an imbalance of the knee is present the soft tissue situation can be rerecorded and a new planning can be made with recutting the bones by 0.5° and 1 mm steps.

Following the robotic-assisted execution of the cuts the prosthesis is implanted manually.

## Case report

As examples two consecutive patients operated on with the robotic-assisted technology are included in this article. Both patients are enrolled in an ongoing prospective randomized controlled trial comparing robotic-assisted TKA with individualized alignment versus conventional manual technique. They gave informed consent to being enrolled in this clinical trial. The study protocol was approved by the local ethics committee. The study is registered in the German Clinical Trial Register under DRKS00012390.

Inclusion criteria are osteoarthritis of the knee with the indications for TKA and a University of California Los Angeles (UCLA) activity scale of more than 4 points. Exclusion criteria are defined as body mass index (BMI) >40, history of infection, osteotomy, or osteosynthesis around the affected knee, chronic rheumatic disease, neurological diseases affecting mobility of the patient or an American Society of Anaesthesiologists (ASA) risk classification of 3 or more. For outcome measurement, the standard 2011 Knee Society score (KSS), Western Ontario and McMaster Universities Osteoarthritis Index (WOMAC) and Oxford knee score (OKS) are collected the day before surgery and at 3 months postoperatively. Major and minor complications or revision surgeries during the follow-up are also recorded. On preoperative and postoperative (5–7 days) long leg standing radiograms standard mechanical tibiofemoral angle (OLA), medial proximal tibial angle (MPTA), and mechanical lateral distal femoral angle (mLDFA) are determined.

## Results and experience

The video demonstrates that a purely anatomical position of the prosthetic components might not automatically lead to perfect soft tissue balance. Also, with different cartilage thickness for example in the medial and lateral compartment, the overall limb alignment is off the physiological orientation, when only a bone scan is taken into account. Therefore, minor adaptations of the prosthesis by approximately 1–2 mm are necessary to perfectly balance the gaps. The cadaver example shown in the video demonstrates that not only these adaptations have great effects on the soft tissue balance but also the center of rotation about which the positional change is made. If the pivot point is in the center of the knee both lateral and medial compartments are affected. If the pivot point is shifted to the already balanced medial compartment, for example, changes of the angulation only influence the contralateral compartment.

**Fig. 4:**
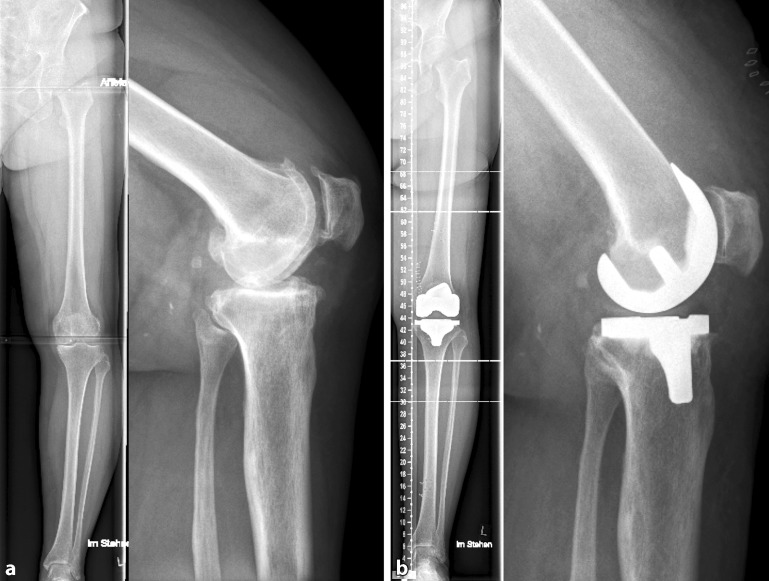
Radiological example on individualized alignment for TKA. **a** Preoperative and **b** postoperative. a. p. standing long leg and sagital weight bearing radiograms

The two individual patients displayed in more detail were 67 and 58 years of age and both presented a high activity level of 7 at the time of surgery. The preoperative alignment, the targeted alignment after adaptation to soft tissue, and the final postoperative radiographic outcome are displayed in Table 1 and with the radiographs shown in Fig. 4. Both patients had a planned alignment off the traditional neutral mechanical axis with an oblique joint line, although both were within the accepted range of 3° deviation to the mechanical axis. A variation between −1.4° and 3.9° external rotation to the TEA was observed with reference to the posterior condylar axis. The reduction of the tibial slope resulted in an adaptation on the posterior femur; however, this was approximately 1 mm on average. A perfect extension gap balance was achieved in each case, whereas the lateral flexion laxity often varied by 1–2 mm (Table 2).

**Table 1:** Preoperative and resulting postoperative alignment as well as the planned alignment parameters for the two patients

Patient ID	Pre-OP OLA	Planned OLA	Post-OP OLA	Pre-OP MPTA	Planned MPTA	Post-OP MPTA	Tibial slope	Pre-OP mLDFA	Planned mLDFA	Post-OP mLDFA	Femoral flexion	Planned rotation TEA
1	6.5	2	1.8	87	89	89	4	90	91	91	3	3.9
2	9.6	3	3.0	85	86	87	3	90	89	90	5	−1.4

All values given in degrees (°), “−” means internal rotation. PostOP OLA measured on long leg standing radiograms at days 5–7 post-surgery with limited weight bearing

*Pre*-*OP* preoperative, *Post*-*OP* postoperative, *MPTA* medial proximal tibial angle, *mLDFA* mechanical lateral distal femoral angle, *OLA* overall limb alignment, *TEA* transepicondylar axis

**Table 2:** Resulting flexion and extension gaps (in mm) in the two patients

Patient ID	Extension gap balance		Flexion gap balance	
	Medial	Lateral	Medial	Lateral
1	18	18	17	18
2	19	19	18	20

Regarding the clinical outcome the greatest improvement was seen in the WOMAC starting at 53 and 20 points for the two patients and going down to 5 and 3 points, respectively (range 0–100 points, 0 is best). Interestingly, both patients reported that the pain level and the activity level were much better than expected at 3 months, and one patient already returned to work within 3 months follow-up. As in other clinical studies the postoperative pain level was relatively low, potentially due to the soft tissue protection with the use of the robotic device.

## Discussion and current evidence

This study reports on the initial experience and outcome of image-based robotic-assisted TKA aiming for patient individualized alignment. The majority of knees that were operated on based on this algorithm were aligned within the accepted range of 3° to the mechanical axis; however, individualized component position is implemented in every case with promising clinical results.

In the varus patients the basic principle of kinematic alignment is followed for restoring the articular surface of the knee with the prosthetic components. This means a purely symmetrically measured resection on the femur according to the thickness of the implant and a corresponding anatomical resection of the tibia plateau; however, KA is often criticized for this purely anatomical approach. First, the resulting soft tissue balance is only a theoretical assumption and later adaptions of the prosthetic position are difficult to control with respect to overall alignment. The great benefit of the presented robotic system is the inclusion of the soft tissue situation into the virtual planning. Thus, adaptions with respect to the soft tissue can be made, while having the effect on the overall alignment visible. The maximum overall varus alignment in the algorithm has been set to 3–4°, as this is still the accepted range. On the tibial cut a maximum of 4° tibial varus is currently accepted. This is based on several papers outlining that larger deviations may lead to a significant increase of the medial compartment load on the tibia with the potential risk of early implant failure [19, 26, 28]. Marchand et al. recently published a retrospective analysis of 330 robotic-assisted TKA cases reporting that they corrected severe deformities in the appropriate direction within a few degrees of neutral and avoiding overcorrection [24]. Even if the physiological joint line obliquity is a slope of more than 4°, the system gives the opportunity to reduce this pathologic situation without affecting the gap width. With the CT-based planning, not only the angulation of the cut on the tibia can be individually adjusted but also the pivot point around which angulation is set. This enables the actual cutting thickness on each compartment to defined more independently from the resulting angle. In this way a MPTA of 85° can be reduced to the accepted range with no need for soft tissue releases, as seen in patient 2. Marchand et al. have also shown that this technique is suitable for difficult cases with severe deformities [23]. A second point of criticism on KA is, that even with resection of the anterior cruciate ligament in TKA, the natural knee kinematics cannot be restored with the prosthesis. There are good arguments to reduce the tibial slope compared to the natural situation to maintain the anteroposterior stability and rollback kinematics of the knee [13, 29]. There is evidence that large tibial slope affects the knee stability and implant longevity [27]. This contradicts the purely anatomical approach regarding the component flexion; however, reducing the tibial posterior slope may affect the flexion gap width and would possibly need adaptation on the posterior femur. In this algorithm, we reduce the tibial slope comparted to the physiological situation to compensate for ACL loss; however, the amount of compensation can be virtually planned and again, the effect of reducing the slope could be adjusted by the surgeon by setting the pivot point for this parameter more anteriorly or posteriorly. This already results in a different effect on the flexion gap; however, it was possible to see that the reduction of the tibial slope leads to larger posterior femoral resection in all cases to achieve the desired gap width. In this context, it has to be mentioned that reduction of posterior condylar offset (PCO) is critical for flexion ability and resulting stability of the knee [25, 29]. Thus, the maximum posterior resection is now set to 9 mm in the algorithm, as Matziolis et al. [25] reported a tight range by only 2 mm deviation. Otherwise, the femur would be shifted distally by 1–2 mm and more lateral laxity would be accepted in order to maintain the a more physiological PCO.

Third, a potential internal rotation of the femur is sometimes criticized even in the kinematic concept, as the femoral rotation is set to the PCL. This may result in a relative internal rotation to the TEA, as seen in patient No. 2; however, in our experience this does not automatically mean an internal rotation to the patient’s individual trochlear orientation and can be visualized on the CT model-based virtual planning. As the femoral rotation is not a major parameter to balance the knee in this algorithm, the necessity to set a limitation on the rotation has not been seen. An internal rotation relative to the native trochlea should potentially be avoided, as most current knee designs have the concept of external rotation implemented in the trochlear geometry.

In summary, in all knees that were initially planned for KA minor adaptions were necessary to achieve the set goal of symmetrically balanced gaps and to account for general biomechanical principles in TKA. Regarding patient outcomes, statements must be made very carefully. This is because of the unrepresentative sample group as well as the still limited overall experience and short follow-up period. Up to now, there is only one publication available comparing the short-term clinical outcome (6 months) of robotic-assisted versus manual TKA of this specific system. As a result, a significantly better outcome in short-term pain, physical function and total satisfaction was seen in favor of the robotic group [22]. A recent review summarized similar effects also for competing technologies [20].

### Pros and cons compared to other technologies

The robotic technology introduced here means relevant technical overheads and additional costs that need to be put into the context of competing approaches. Compared to traditional navigation the major disadvantage is the higher costs for the system, technical support and a longer set-up time prior to surgery. Also, the necessity of a preoperative CT scan results in extra cost; however, the CT-based bone model utilizes a higher precision in the planning and intraoperative navigation and in addition, the robot means an active real-time control on the definite cuts whereas in navigation these are only passively checked after the cuts have been made and the robot enables active soft tissue protection that has also been shown in in vitro studies. Compared to patient-specific instruments (PSI templates) there is the same need for a preoperative CT scan. In theory the same planning algorithms can be conducted as introduced in this article; however, in the PSI technology there is no information on the soft tissue and no virtual intraoperative adaptations can be made. Moreover, the segmentation and prosthesis planning is made on side in the hospital and thus can be much better controlled by the surgeon than in PSI.

The intraoperative time is comparable to the manual standard procedure or navigation, where most time is needed for the navigation part and the virtual planning. Once the plan is approved the time for cutting the bones with the robotic device is less compared to conventional instrumentation as no cutting guides are needed. A precise cost and time analysis comparing these approaches is currently being carried out in a multicenter study.

## Conclusion

Based on initial experiences with image-based robot-assisted TKA this technology is judged to enable the surgeon to overcome the limitations associated with standard mechanical alignment in a controlled and objective manner. With the combination of an anatomical approach to preposition the components and the adaptation of this planning with respect to soft tissue tension, it is possible to create an individualized, optimized alignment with symmetrical gap balance and a reduced need for soft tissue release. The current clinical results, in this study as well as the literature, are promising but only a very short follow-up is currently available. Further studies and longer follow-up are necessary. Critical success factors are the underlying algorithms to balance and adjust the components that are discussed in detail here. This is the starting point for further evaluation and optimization.

## Caption Electronic Supplementary Material

Video 1 Patient individualized alignment in total knee arthroplasty using image-based robotic assistance (with kind permission of © T. Callies, all rights reserved)

## Abbreviations

ACL: Anterior cruciate ligament
ASA: American Association of Anaesthesiologists
KA: Kinematic alignment
KSS: Knee Society score
MA: Mechanical alignment
mLDFA: Mechanical lateral distal femoral angle
MPTA: Medial proximal tibial angle
OKS: Oxford knee score
OLA: Overal Limb alignment (mechanical tibiofemoral angle)
PCL: Posterior cruciate ligament
PCO: Posterior condylar offset
TEA: Transepicondylar axis
TKA: Total knee arthroplasty
WOMAC: Western Ontario and McMaster Universities Osteoarthritis Index
